# The Red Imported Fire Ant (*Solenopsis invicta* Buren) Kept Y not F: Predicted sNPY Endogenous Ligands Deorphanize the Short NPF (sNPF) Receptor

**DOI:** 10.1371/journal.pone.0109590

**Published:** 2014-10-13

**Authors:** Prati Bajracharya, Hsiao-Ling Lu, Patricia V. Pietrantonio

**Affiliations:** Department of Entomology, Texas A&M University, College Station, Texas, United States of America; Medical School of Hannover, Germany

## Abstract

Neuropeptides and their receptors play vital roles in controlling the physiology and behavior of animals. Short neuropeptide F (sNPF) signaling regulates several physiological processes in insects such as feeding, locomotion, circadian rhythm and reproduction, among others. Previously, the red imported fire ant (*Solenopsis invicta*) sNPF receptor (*S. invicta* sNPFR), a G protein-coupled receptor, was immunolocalized in queen and worker brain and queen ovaries. Differential distribution patterns of *S. invicta* sNPFR protein in fire ant worker brain were associated both with worker subcastes and with presence or absence of brood in the colony. However, the cognate ligand for this sNPFR has not been characterized and attempts to deorphanize the receptor with sNPF peptides from other insect species which ended in the canonical sequence LRLRFamide, failed. Receptor deorphanization is an important step to understand the neuropeptide receptor downstream signaling cascade. We cloned the full length cDNA of the putative *S. invicta* sNPF prepropeptide and identified the putative “sNPF” ligand within its sequence. The peptide ends with an amidated Tyr residue whereas in other insect species sNPFs have an amidated Phe or Trp residue at the C-terminus. We stably expressed the HA-tagged *S. invicta* sNPFR in CHO-K1 cells. Two *S. invicta* sNPFs differing at their N-terminus were synthesized that equally activated the sNPFR, SLRSALAAGHLRYa (EC_50_ = 3.2 nM) and SALAAGHLRYa (EC_50_ = 8.6 nM). Both peptides decreased the intracellular cAMP concentration, indicating signaling through the G_αi_-subunit. The receptor was not activated by sNPF peptides from other insect species, honey bee long NPF (NPY) or mammalian PYY. Further, a synthesized peptide otherwise identical to the fire ant sequence but in which the C-terminal amidated amino acid residue ‘Y’ was switched to ‘F’, failed to activate the sNPFR. This discovery will now allow us to investigate the function of sNPY and its cognate receptor in fire ant biology.

## Introduction

Neuropeptides and their receptors play critical roles in almost every aspect of insect life [1]. Therefore, they are of great interest to researchers in insect neurobiology and physiology and also to those interested in target validation for pesticide discovery and pest management. The red imported fire ant (*Solenopsis invicta* Buren) is a eusocial insect species native to South America. Because of its extraordinary capacity to adapt to different environmental conditions and its aggressiveness, the species is invasive and successfully establishes affecting the habitat of native animals; this has occurred in the United States, Australia, New Zealand, mainland China and Taiwan [2]–[5]. *S. invicta* colonies can recover even after destruction of 90% of their workers [6] making their management very difficult. In addition to their high reproductive output and aggressiveness, fire ants are an interesting model for the study of social organization and behavior because colonies are present in monogyne (single queen) and polygyne (multiple queens) forms [7], [8].

We are systematically studying fire ant receptors involved in critical physiological functions, especially signaling receptors, to gain knowledge of physiological mechanisms in fire ants that perhaps collectively contribute to their success, life history, high reproductive output and colony growth. The neuropeptide Y (NPY) signaling system is recognized as evolutionarily conserved in animals and regulates several physiological processes such as feeding behavior, obesity, stress, blood pressure, anxiety, memory and circadian rhythms [9]–[14]. Vertebrate NPY is structurally and functionally related to the NPF family in invertebrates [15]–[19]. In insects, the neuropeptide F (NPF) peptide family is represented by two forms, the long NPF (NPF) and short NPF (sNPF) [20]. NPFs are commonly 28–45 amino acids long with C-terminal conserved sequences being RxRFamide or GRxRYamide, while sNPFs are the short peptide sequences of 6–19 amino acid residues ending in RFamide or RWamide [21].

sNPF has been identified exclusively in arthropods [21]. sNPF peptides have been isolated from several insect species including the Colorado potato beetle [22], the American cockroach [23], the desert locust [24], and the fruit fly [25]. The peptide predicted sequence has also been identified by genomic and EST analyses in fruit fly and mosquito [25], [26]. In most of the species, a single sNPF gene encodes multiple sNPF isoforms [27]–[31], whereas in the honey bee the single sNPF gene encodes a single form of sNPF [32].

In solitary insects, sNPF is involved in various insect physiological functions such as locomotion, circadian rhythms, reproduction and feeding behavior [20], [33]–[35]. In *Drosophila* sNPF is also involved in the regulation of sleep homeostasis through the modulation of the cAMP-PKA-CREB signaling pathway [36], [37]. sNPF seems to have a species specific effect in feeding behavior and several studies indicate the positive relationship of activation of the sNPF signaling system (increased expression) and food searching and feeding behavior. In *Drosophila,* the over expression of sNPF increases adult body size and the number of feeding flies [35], [38]. Further, in adult flies, expression of sNPF in olfactory receptor neurons mediates the enhancement of starvation-dependent food-searching behavior [39]. Starvation increases expression of sNPF in cockroach [40] and sNPF receptor (sNPFR) in honey bee [41]. Transcript levels of the sNPF and cognate receptor in the honey bee, are interpreted as associated with worker division of labor and nutritional status, because increases in both transcripts were observed in foragers from nutritionally poor colonies [41]. Peptidomics of the honey bee (*Apis mellifera*) brain identified the sNPF abundance changed in association with nectar and pollen foraging [42]. In other insect species such as the lepidopteran silkworm, *Bombyx mori*, and the red imported fire ant queens, sNPF and sNPFR transcript levels, respectively, decrease with food-deprivation, pointing to a negative correlation of sNPF activation and fed status. Similarly, an inverse relation of sNPF signaling and feeding behavior was also observed in the desert locust. Injection of sNPF peptides decreases food uptake while knock down of sNPF prepropeptide and sNPFR genes stimulates it [27], [28]. Some of these generalizations, however, are made on the basis of transcript levels alone, and not peptide and/or receptor protein, so caution is needed when interpreting them.

In fire ant, the full length cDNA of the sNPFR was originally cloned from queen ovaries [43]. Detection of the sNPFR proteins both in brain and in oocytes of fire ant queens [44] strongly suggests a link between feeding and reproductive functions of sNPF in fire ants. A reduction of sNPFR transcript expression was observed in starved mated queen brain, implying a negative relationship between sNPFR transcript expression and fed status [43]. It must be noted that these queens were only given water and they had been isolated from the colony. In workers of the fire ant, sNPFR immunoreactive cells in the brain were higher in number in the minor subcaste (workers that were involved in queen and brood care), followed by medium and major worker subcastes. In addition, the number of sNPFR expressing neurons changed in the worker brain, decreasing in all worker subcastes when brood was absent. It was speculated that higher number of sNPFR immunoreactive neurons in the presence of brood may reflect the detection of protein, or the need for protein when larvae of the 4^th^ instar, which digest protein actively for the rest of the colony, are present [45].

sNPFRs have been functionally characterized only in solitary insects, flies (mosquitoes and fruit fly) and locust [25], [27], [46]. For this study our attempts to deorphanize the sNPF receptor using the orthologous peptide from the honey bee and those from *Drosophila* failed: none of these activated the receptor. We thus cloned the full length cDNA of the elusive sNPF ligand. Unexpectedly, the fire ant sNPF peptide ends with ‘Y’ (tyrosine), differing from other known insect sNPF sequences ending in F (phenylalanine) or W (tryptophan). Two predicted peptides, when C-terminally amidated, activated the *S. invicta* recombinant sNPFR, which behaves as a Gi-coupled receptor in CHO-K1 cells, decreasing intracellular cAMP. Ligand identification will allow testing new hypothesis on the role of sNPY in social organization and overall fire ant biology.

## Materials and Methods

### Fire ant collection and tissue isolation

Newly mated, dealate, fire ant queens were collected after mating flights in the campus area of Texas A&M University, College Station, Texas. Queens were abundantly found around 4–5 PM the day after a heavy rain when sunny and warm conditions were present, as is known [8], [47]. After collection, queens were placed in humidified tubes until dissection, as described [48]. Whole brains were dissected in phosphate buffer saline (PBS) under a dissection microscope and kept in RNAlater solution (Ambion, Life Technologies, Carlsbad, CA, USA) at −20°C until use.

### RNA isolation

Total RNA and mRNA were isolated to synthesize two different cDNA types. For total RNA isolation from fire ant brain, RNAlater solution was removed and 1 ml of TRIzol reagent (Invitrogen, Life Technologies) was added. Brains were homogenized with a disposable polypropylene pestle homogenizer (VWR, Radnor, PA, USA); chloroform (200 µl) was added to the homogenate and vortexed for 15 s. The chloroform/TRIzol reagent mixture was incubated at room temperature (RT) for 10 min followed by centrifugation at 12,000 g at 4°C for 10 min. The total RNA pellet was obtained by conventional isopropanol/ethanol precipitation, washed in 70% ethanol, air dried briefly and dissolved in nuclease free water and stored at −80°C until use.

mRNA was isolated from the newly mated fire ant queen brains using the DynaBeads mRNA direct kit (Invitrogen) following the manufacturer’s protocol. In brief, RNAlater solution was removed from the tubes containing brains, and 500 µl of lysis/binding buffer was added. After tissue homogenization, additional lysis/binding buffer was added up to 1 ml. The Dynabead solution (250 µl) was placed in an Eppendorf-tube and washed with lysis/binding buffer. Tissue homogenate was mixed with the Dynabeads and incubated with continuous mixing at RT for 10 min to allow binding of the poly (A) tail of the mRNA to the bead-oligo(dT)_25_. After incubation, the tube was placed on the magnet for 2 min and the supernatant was removed. The beads/mRNA complex was washed two times with 500 µl of washing buffer A provided with the kit at RT, followed by washing with buffer B once. The magnet was used to separate the beads-mRNA from the washing buffers in each washing step. Elution of mRNA from the beads was by incubating bead/mRNA complex with 19 µl of 10 mM Tris-HCl (elution buffer) at 75°C for 2 min. RNaseOUT recombinant ribonuclease inhibitor (Invitrogen) (1 µl) was added to the collected mRNA to avoid mRNA degradation. The mRNA was stored at −80°C until use.

### cDNA synthesis

To clone the cDNA for the sNPF, cDNA was synthesized from 0.5 µg of total RNA using SuperScript III First-Strand Synthesis System (Invitrogen) following the manufacturer’s specification. Oligo(dT)_20_ (1 µl of 50 µM) and random hexamers (1 µl of 50 ng/µl) were added to the total RNA. DEPC-treated water was added to 10 µl and the mixture was incubated at 65°C for 5 min and chilled on ice. A reverse transcription reaction master mix containing 2 µl of 10×RT buffer, 4 µl of 25 mM MgCl_2_, 2 µl of 0.1 M DTT, 1 µl of RNase OUT (40 U/µl) and 1 µl of SuperScript III reverse transcriptase (200 U/µl) was added, mixed and centrifuged. The mixture was incubated at 25°C for 10 min followed by 50°C for 1 h and the reaction was terminated at 85°C for 5 min and chilled on ice. The synthesized cDNA was incubated with 1 ul of RNase H at 37°C for 20 min and stored at −20°C until use. For cloning of the 5′ and 3′ region of the sNPF peptide cDNA sequence, RACE ready cDNA was synthesized from 11 ng of mated queen brain mRNA using the SMARTer RACE cDNA Amplification Kit (Clontech laboratories, Mountain View, CA, USA) following the manufacturer’s protocol. The synthesized RACE ready cDNA (10 µl) was stored at −20°C until use.

### Cloning of *S. invicta* sNPF

The honey bee sNPF prepropeptide sequence [32] was blasted against the ant genome portal (http://hymenopteragenome.org/ant_genomes/). This blast search identified other similar ant sequences from *Camponotus floridanus, Harpegnathos saltator*, *Acromyrmex echinatior* and *Solenopsis invicta*. Alignment of these sequences revealed divergence for the *S. invicta* predicted peptide region. This was initially suspected as perhaps a sequencing error and we sought access to the assembled RNA sequence, which facilitated the sNPF cloning process (RNAseq data provided by Yannick Wurm, Hugh Robertson, Oksana Riba-Grognuz and Laurent Keller). To clone the central region of the sNPF cDNA, forward (*Si* sNPF-F1) and reverse (*Si* sNPF-R1) specific primers were designed (Table S1). The initial fragment of sNPF was amplified from cDNA template with primers *Si* sNPF-F1 and *Si* sNPF-R, following the PCR condition as: 94°C for 5 min followed by 40 cycles of 94°C 30 s, 64°C 30 s and 72°C 3 min. To obtain the 5′ and 3′ region of the sNPF, three specific primers (Table S1) were designed for the amplification using the 5′- and 3′- RACE ready cDNA as the templates, respectively. Conditions of RACE PCR were as follows: 5 cycles of 94°C 30 s and 72°C 3 min followed by 5 cycles of 94°C 30 s, 70°C 30 s and 72°C 3 min, and 40 cycles of 94°C 30 s, 68°C 30 s and 72°C 3 min. PCR products were analyzed by agarose gel electrophoresis and bands were cut and cloned using the TOPO TA cloning kit (Invitrogen) and sent for sequencing at the Gene Technologies Laboratory (Texas A&M University, College Station, TX, USA).

### Sequence and phylogenetic analyses

**Figure 1:**
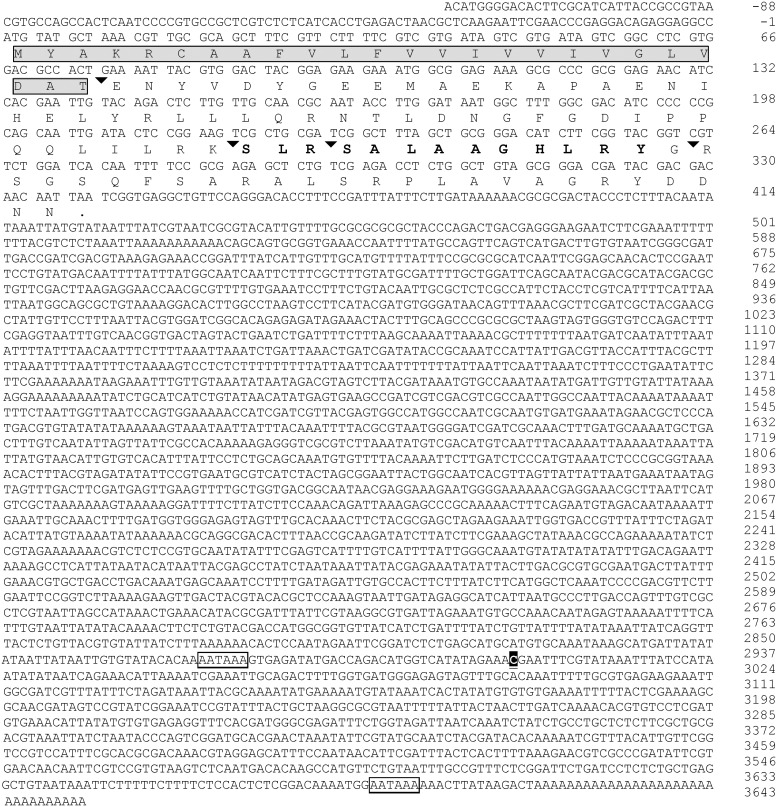
Nucleotide and deduced amino acid sequence of *Solenopsis invicta* short neuropeptide F (GenBank accession number KJ812404). Arrowhead indicates the predicted post-translational cleavage site for processing the prepropeptide to active peptide(s). The predicted signal peptide is shaded in gray and boxed, the predicted active peptide sequence is in bold and the polyadenylation signal sequences in the 3′UTR are boxed [49]. Poly (A) tail of the shorter cDNA begins after nucleotide ‘C’ shaded black.

The cDNA sequence obtained was analyzed with the Lasergene software (DNASTAR, Madison, WI, USA) and the prepropeptide open reading frame (ORF) was predicted by the “ORF finder” tool in the same. In addition, the initiation site (AUG) was also confirmed through analysis using ATGpr (atgpr.dbcls.jp) that identified the Kozak consensus sequence. Detection of poly (A) signal sequences by the poly(A) signal miner http://dnafsminer.bic.nus.edu.sg/PolyA.html [49]. The BLAST search algorithms at NCBI were used to identify the sNPF prepropeptide sequences from other insect species and alignment of the *S. invicta* sNPF prepropeptide to those was with ClustalW included in Megaline (Lasergene, DNASTAR). The sNPF prepropeptide sequence was analyzed with the prediction server (cbs.dtu.dk/services/SignalIP/) to determine the signal peptide sequence. Subsequently, to determine the first cleavage site for the active peptide sequence, a sequence of two basic amino acids (RK) was localized by eye-gazing in the propeptide because these are the common cleavage sites in insect neuroendocrine peptides [24]. However, Southey et al. [50] have also identified ‘R’ in addition to ‘RK’ as a potential cleavage site. The sNPF sequence has an ‘R’ residue downstream of RK, which could also be a first cleavage site. To determine the C-terminal sequence the alignment to other peptides and the orthologous neuropeptide of *Apis mellifera* sequence were also compared to the *S. invicta* sNPF sequence. Based on these comparative analyses and the amidation signal at the C-terminus, the putative active peptides were predicted as SLRSALAAGHLRYa (sNPF1) or SALAAGHLRYa (sNPF2) (Figure 1). The evolutionary relationships of the insect sNPF prepropeptide amino acid sequences were analyzed by MEGA (version 5.05) [51] and neighbor-joining method with 1000 bootstrap replicates. The resulting tree was exported to Newick and then to FigTree 1.4.0 (http://tree.bio.ed.ac.uk/software/figtree/) for creating the unrooted phylogenetic tree figure.

### Matrix assisted laser desorption ionization time of flight mass spectrometry (MALDI-TOF MS)

To identify the endogenous sNPF peptides, 0.3 g in total of eggs, and larvae and pupae (brood) were collected from a laboratory maintained mature polygyne colony. These were homogenized and sonicated in 500 µl of a methanol: water: trifluoroacetic acid (TFA) solution (90∶9∶1). The homogenate was centrifuged at 9,600 g, 4°C for 10 min. The homogenization, sonication and centrifugation were repeated. The supernatant was pooled with the previously collected supernatant. Peptides were isolated according to Boerjan et al. [52], except that an equal amount of hexane was then added to the latter homogenate to remove the lipid. This mix was vortexed and centrifuged at 9,600 g, 4°C for 10 min. The lipid-free aqueous phase was collected for MALDI-TOF MS. The samples were concentrated and desalted (ZipTip C_18_, Millipore, Billerica, MA). The concentrated sample (1 µl) was placed on a MALDI steel plate and covered by the matrix (5 mg/ml of Alpha-Cyano-4-hydroxycinnamic acid in 70∶30 acetonitrile: water). After air drying the matrix for a few seconds, the sample was analyzed by MALDI-TOF MS with an Applied Biosystems 4800 TOF Analyzer (AB SCIEX, Framingham, MA, USA) at the Laboratory for Biological Mass Spectrometry, Department of Chemistry, Texas A&M University, College Station, TX, USA.

### Establishment of stable CHO-K1 cell line expressing the fire ant sNPF receptor

Originally, the fire ant sNPFR (*S. invicta* sNPFR) cDNA (GenBank AAY88918.1) was cloned from queen ovary in 2006. Upon re-sequencing of this clone and comparison with the submitted sequence we discovered typing errors in the GenBank sequence [the fifth residue was reported as D (GAC) while the correct residue is N (ACC), the 42^nd^ residue was V (GTG), the correct is M (ATC), and the 456^th^ nucleotide as C, while the correct nucleotide is T]. The correctness of the sequence was also reconfirmed by additional amplification, cloning and sequencing of the sNPFR ORF from nine brains of newly mated queens. The correct sequence of the receptor expressed in CHO-K1 cells herein is reported as supplementary information (Figure S1).

For expression of this receptor cDNA in CHO-K1 cells, the clone designated p*Si*sNPFR#16 (in the TOPO vector pCR2.1, Invitrogen) containing the full-length cDNA of the *Si*sNPFR was used as the template for a PCR to add a nine amino acid residue haemaglutinin tag (HA-tag; YPYDVPDYA) at the receptor N-terminus, following published procedures [53]–[55]. For this, three overlapping forward primers were designed (Table S1). The first forward primer (primer #129), includes a *Xho*I restriction site for directional cloning, the Kozak sequence, a start codon, and a partial sequence of the HA-tag. The second primer (sNPFR-f1) contains the full HA-tag sequence. The third primer (sNPFR-f2) contains the terminal HA-tag sequence and a *S. invicta* sNPFR sequence corresponding to the N-terminus. A reverse primer (sNPFR-r1) was designed with an *Eco*RI restriction site for directional cloning. The three forward primers and the reverse primer were simultaneously used in a PCR amplification reaction. The concentration of the second and the third 5′ primers (4 nM for each) was 1% of that of the first forward primer (400 nM) to favor the synthesis of the longest, complete product. The reverse primer was at the same concentration (400 nM) as the first forward primer. PCR amplifications contained 350 ng template plasmid, the four mentioned primers, 0.2 mM of dNTPs, 1x Advantage 2 buffer and 1 µl Advantage 2 Taq DNA polymerase (Clontech) in a final volume of 50 µl. The PCR settings were as follows: 94°C for 3 min; followed by 40 cycles: 94°C for 30 s, 65°C for 1 min, 72°C for 1.5 min; 72°C extension for 10 min. The PCR product was digested with *Xho*I and *Eco*RI (Promega, Madison, WI, USA) and the band was recovered after gel electrophoresis. The retrieved *Xho*I-*Eco*RI fragment DNA was ligated with T4 DNA ligase (Promega) to the linearized pcDNA3.1(-) vector (Invitrogen) previously digested with *Xho*I and *Eco*RI. The ligation mix was transformed into One Shot Top 10F’ *E. coli* competent cells (Invitrogen). Positive clones were identified by restriction analysis of recombinants with *Xho*I and *Eco*RI and gel electrophoresis. The correctness of the expression plasmid (pcDNA3.1(-) *SisNPFR*) for receptor expression was confirmed by sequencing.

For the functional characterization of *S. invicta* sNPFR, stably transformed cell lines were established as described [56]. In brief, CHO-K1 cells were obtained from the American Type Culture Collection (ATCC, Manassas, VA, USA) and grown as described [57] in a 5% CO_2_ humidified incubator at 37°C. For transfection, CHO-K1 cells were seeded into T-25 tissue culture flasks and allowed to grow overnight until 50% confluent. Cells were transfected in serum-free Opti-MEM medium (Life Technologies, Gaithersburg, MD, USA) with the cationic lipid reagent Lipofectin (Life Technologies) (6 µl) and 2 µg of the expression construct according to the manufacturer’s protocol. After 6 h, the lipofectin-containing medium was removed and replaced with F12K (Sigma, St. Louis, MO, USA) medium plus 10% fetal bovine serum (EquiTech Bio, Kerrville, TX, USA) without antibiotic. After 48 h of growth, the cells were split into the same medium but with antibiotic Geneticin (Invitrogen) (800 µg/ml), and selection continued for 5 weeks followed by isolation of clonal lines. A total of 14 clonal cell lines were obtained by dilution and selection of single cells distributed in a 96-well plate (96 theoretical single cells). Presence of *S. invicta* sNPFR transcript was verified by screening by RT-PCR by pooling aliquots from clonal cell lines. For this, total RNA was isolated by RNeasy Mini kit (Qiagen) and used for cDNA synthesis with SuperScript III First-Strand Synthesis System followed by RT-PCR with receptor forward sNPFRf1 and reverse sNPFRr1 primers (Table S1). The cell lines from groups that generated the RT-PCR product of the expected size were further screened. Single positive clonal cell lines were subjected to functional assay using *S. invicta* sNPF1 and sNPF2 as ligands. Cell line C6 was further selected to obtain a line designated *Si*sNPFR-C6E8, which was the best performing cell line and was chosen for receptor functional characterization.

### Immunocytochemistry of *Si*sNPFR-C6E8 cell line to detect HA-tagged sNPF-receptor

For immunocytochemistry experiments, 1×10^5^ cells were sown over sterile circular coverslips (Fisher Scientific, Pittsburgh, PA, USA), that were placed in, 12-well plates. Cells, mock-transfected with vector pcDNA3.1(-) only, and *Si*sNPFR-C6E8, were allowed to attach onto the coverslip after incubation, for 24 h. Cells were then processed for immunolocalization of the HA-tag-labeled receptor according to methods in Yang et al. [55]. Briefly, cells were prefixed in freshly prepared 4% paraformaldehyde (PFA) in F12 K culture medium with 10% fetal bovine serum and Geneticin. This prefixative solution was removed and then cells were washed with PBS and fixed with 4% PFA for 15 min. Fixed cells were permeabilized in PBST (0.25% Triton X-100 in PBS) for 5 min, washed with PBS and covered with Image-IT FX signal enhancer (Invitrogen). The plate was incubated in a humid chamber for 30 min. Cells were then incubated in blocking solution [5% normal goat serum (NGS; Jackson ImmunoResearch, West Grove, PA, USA) in PBS] at 4°C, overnight. Then, cells were stained with a rabbit antiHA-tag primary antibody (Cell Signaling Technology, Danvers, MA, USA) solution (0.23 µg/ml) in 2% NGS in PBS at 4°C, overnight. The cells were washed with PBS and incubated with goat anti-rabbit IgG Alexa Fluor 546 (10 µg/ml) (Jackson ImmunoResearch) at 4°C, overnight. Cover slips were placed onto slides and mounted with VectaShield containing DAPI (Burlingame, CA, USA). *Si*sNPFR-C6E8 cells were incubated with preimmune rabbit serum (1∶2000) as negative controls, and also incubated with rabbit anti α-tubulin IgG (1∶50 dilution; Abcam, Cambridge, MA, USA) as a positive control for immunostaining. As negative and positive controls for the HA-tag antibody staining, mock-transfected cells and RmCAP2bCCL#19 [55] cell line expressing an HA-tag neuropeptide receptor were incubated with the anti HA-tag primary antibody, respectively.

### Cell membranes preparation and western blot analysis

A cell membrane enriched preparation of *Si*sNPFR-C6E8 cells was obtained as reported [44]. Briefly, *Si*sNPFR-C6E8 cells were grown in a T-75 flask and once they were 90–95% confluent, the culture medium was discarded and cells were washed with PBS. The cells were harvested using a cell scrapper on 1 ml of buffer A (25 mM Tris HCl, pH 7.5, 1 mM EDTA, 1 mM EGTA, 1 mM DTT) with 1×*cOmplete* protease inhibitor cocktail tablets (Roche Diagnostics Ltd, Mannheim, Germany). All the following steps were performed on ice. The cells were homogenized and the cell nuclear fraction was removed by centrifugation at 800 g at 4°C for 5 min. The supernatant containing soluble cytosolic and insoluble membrane fractions was collected. Homogenization was repeated by adding 500 µl of buffer A, followed by centrifugation and collection of the supernatant. The process was repeated for a total of four times. The final collected supernatant was centrifuged at 100,000 g at 4°C for 1 h. The supernatant with soluble cytosolic protein was discarded and the membrane-containing pellet was dissolved in 200 µl of buffer B (50 mM Tris/HCl, pH 7.5, 2 mM CaCl_2_) with 1×*cOmplete* protease inhibitor cocktail. Protein was quantified by the BCA (Pierce, Rockford, IL, USA) method and stored at −80°C until use.

For western blot analysis, membrane proteins (50 µg) from *Si*sNPFR-C6E8 cells and mock (vector pcDNA3.1(-) only) transfected cells and mated queen ovary were resolved by SDS-PAGE in a 10% Mini-PROTEAN TGXTM precast gels (Bio-Rad Laboratory, Hercules, CA, USA) and transferred onto 0.2 µM Immun-Blot PVDF membrane (Bio-Rad Laboratory) [44]. To block unspecific binding, the PVDF membrane was incubated with 5% skim milk (Walmart) in Tris buffer saline with 0.1% tween 20 (TBST) for 1 h, at RT. After rinsing three times with TBST, the membrane was incubated with 0.4 µg/ml of affinity-purified rabbit polyclonal anti-sNPF receptor antibody [44] at 4°C, overnight. The unbound primary antibody was washed from the membrane with TBST three times for 10 min each. The membrane was then incubated with HRP-conjugated goat anti-rabbit IgG (1∶40,000) (Jackson ImmunoResearch) for 1 h, at RT. The membrane was washed with TBST three times for 10 min each. The membrane was then incubated with SuperSignal West Pico Chemiluminescent substrate (Pierce) for 5 min, exposed to Kodak X-OMAT LS film (Carestream Health, Rochester, NY) and developed with a Konica medical film processor (Konica Corporation, Japan).

### Peptide Synthesis

The sNPF predicted peptides SLRSALAAGHLRYa and SALAAGHLRYa, and an analogous peptide SLRSALAAGHLRFa were commercially synthesized (Genscript, Piscataway, NJ, USA). In addition to these, *Drosophila* sNPF peptides (AAN11060.1), *Drome* sNPF1 and *Drome* sNPF2 were kindly provided by Dr. Joe W. Crim (Department of Cellular Biology, University of Georgia, Athens, GA, USA), and *Drome* sNPF2 12–19 was received as a gift from Dr. Liliane Schoofs (Department of Biology, KU Leuven, Belgium). Honey bee (*A. mellifera*) NPY (NP_001161192.1) was commercially synthesized (Lifetein, NJ, USA) and mouse PYY (AAH10821.1) was purchased from Tocris Bioscience (UK).

### Intracellular measurement of cAMP

To quantify changes in intracellular cAMP, 1×10^5^ cells of *Si*sNPFR-C6E8 were seeded in each well of a 6-well plate and incubated at 37°C in presence of 5% CO_2_ for 24 h to allow cell attachment. Cell culture medium [F12 K medium with 10% fetal bovine serum and 400 µg/ml of Geneticin] was then replaced with the same fresh medium as above and the cells were grown for additional 48 h until they reached 90–100% confluence. The cells were rinsed twice with F12 K medium only (serum and antibiotic free) and then incubated in 50 µM Ro-20-1724 phosphodiesterase inhibitor (Santa Cruz Biotechnology, Santa Cruz, CA, USA) in F12 K medium. Forskolin (10 µM) (Cayman Chemical, Ann Arbor, MI, USA) or forskolin (10 µM) with 0–10 µM solutions of ligands (10 concentrations), respectively, were added to the cells and incubated at 37°C in the presence of 5% CO_2_ for 1 h. Cells were rinsed once with F12 K medium, serum- and antibiotic-free, followed by lysis with 274 µl of 0.1 M HCl at RT for 20 min and centrifugation at 1000 g for 10 min. The supernatant was used for cAMP measurements using the cyclic AMP EIA Kit (Cayman Chemical) following the manufacturer’s protocol. In brief, 50 µl of supernatants and cAMP standards were loaded in a 96-well plate, and the cAMP EIA buffer was used as blank. cAMP AChE tracer (50 µl) (cAMP bound to acetylcholinesterase for competition) and 50 µl of anti-cAMP specific antiserum were added. Then the mixture was incubated at 4°C for 18 h. The mixture was removed and the plate was rinsed five times with wash buffer containing 0.05% polysorbate-20. After washing, the plate was developed at RT for 2 h using the Ellman’s reagent and the OD (412 nm) was measured in a VersaMax tunable microplate reader (Molecular Devices, Sunnyvale, CA, USA). cAMP level was calculated using Cayman provided formulas and aided by software located at http://www.myassays.com/(MyAssays Ltd., Haywards Heath, West Sussex, UK) for the output standard line graph and cAMP concentration calculations. In brief, the standard curve as “Percentage (standard bound/maximum bound) [(%). (B/B_0_)] versus log concentrations” was created and a linear regression fit was performed. For this, the average absorbance readings for non-specific binding (NSB) and B_0_ wells were obtained, and the NSB average was deducted from the B_0_ average to generate the corrected B_0_ maximum binding value. For the remaining standards’ wells, the B/B_0_ value was calculated by subtracting the average NSB absorbance from the standards’ absorbance and dividing the obtained value by the corrected B_0_. To obtain (%). (B/B_0_) the obtained value was multiplied by 100. The cAMP concentration of each sample was determined on the basis of the standard curve. Concentration-response curves were obtained by nonlinear regression-curve fit analysis (sigmoid dose–response equation with variable slope) using Prism 5.0 (GraphPad software Inc., San Diego, CA, USA). Normalized maximal responses from individual replicates at each of 10 concentrations from 0 to 10 µM were used for calculation of the EC_50_s. The percentage of cAMP production for each treatment was expressed as that of the control maximal forskolin cAMP stimulation, and values were obtained from three independent biological replicates, each of which was the average of three technical replicates (pseudoreplicates). The data was statistically analyzed by one-way analysis of variance (ANOVA) followed by Tukey’s multiple comparison test, included in the Prism 5.0 software. To compare the activity of the analogous peptide (SLRSALAAGHLRFa) assays were run similarly except that only two concentrations were tested (10 nM and 1 µM).

## Results

### Cloning of *S. invicta sNPF* and prediction of the active peptide(s)

**Figure 2:**
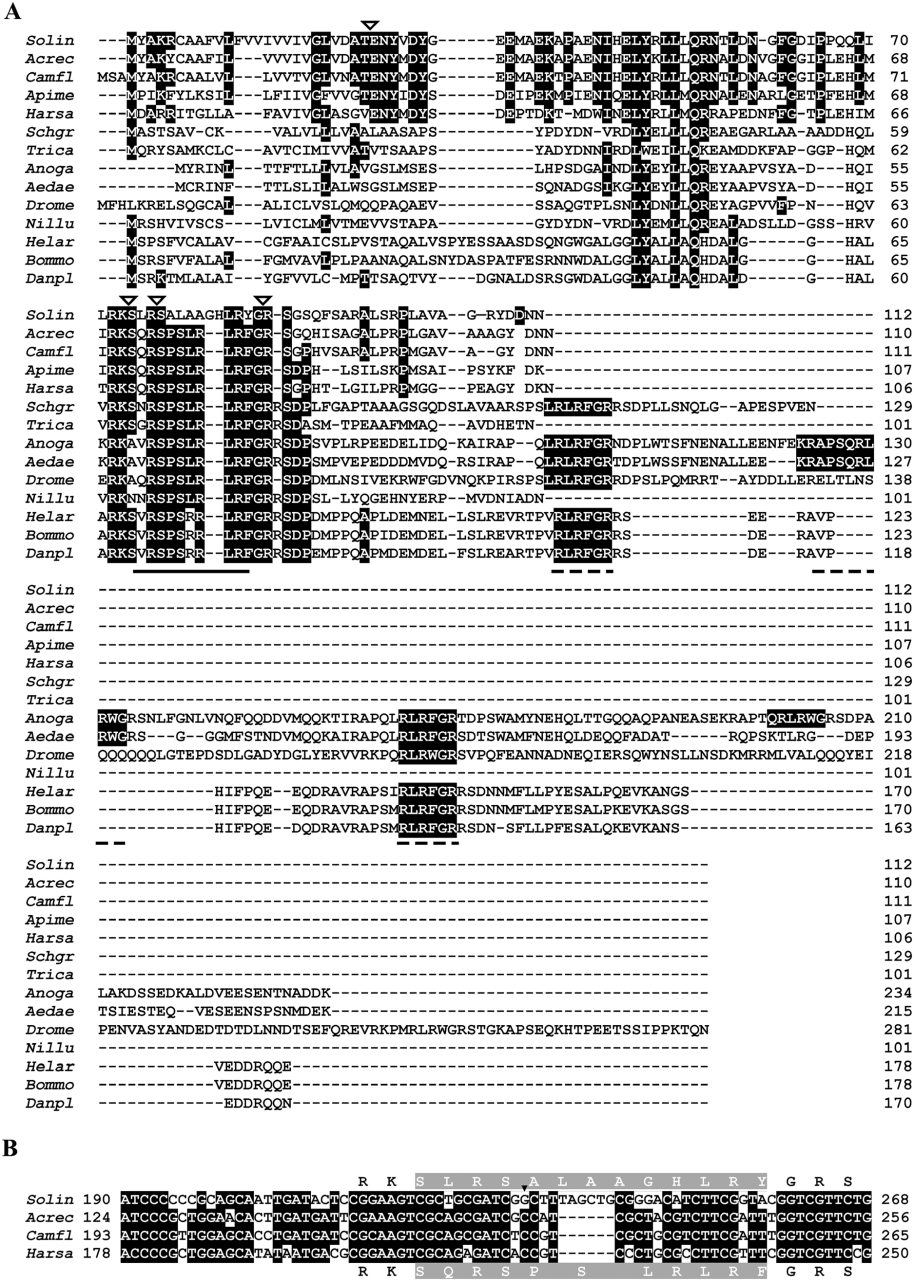
Alignment of insect sNPF sequences. **A.** The unique amino acid sequence of the *S. invicta* short neuropeptide F active peptide(s) is compared to those of other insect species. Aligned sNPF sequences are from (top to bottom): three ant species *Solin* (*Solenopsis invicta* KJ812404), *Acrec* (*Acromyrmex echinatior* EGI59536.1), and *Camfl* (*Camponotus floridanus* EFN66516.1); the honey bee, *Apime* (*Apis mellifera* XP_003250155.1); a basal ant, *Harsa* (*Harpegnathos saltator* EFN85447.1); the locust, *Schgr* (*Schistocerca gregaria* AHH85823.1); the flour beetle, *Trica* (*Tribolium castaneum* EEZ97763.1); dipterans, *Anoga* (*Anopheles gambiae* ABD96048.1), *Aedae* (*Aedes aegypti* ABE72968.1), and *Drome* (*Drosophila melanogaster* AAN11060.1); the brown planthopper, *Nillu* (*Nilaparvata lugens* BAO00976.1), and three lepidopterans, *Helar* (*Helicoverpa armigera* AGH25568.1), *Danpl* (*Danaus plexippus* EHJ63336.1), and *Bommo* (*Bombyx mori* NP_001127729.1). Identical amino acid residues are shaded black. In the alignment, active peptides corresponding to the location of *S. invicta* sNPFs are underlined with a solid line, and additional orthologous peptides encoded in cDNAs from other species are underlined with dashed lines. Arrowheads indicate the predicted post-translational cleavage sites for processing the prepropeptide to active peptide(s). **B.** Alignment of partial sNPF nucleotide sequences encoding the active peptide in four ant species. Identical nucleotides are shaded black. Amino acid residues for the active sNPF peptide (shaded gray) are in the middle of the respective codon; for *S. invicta* are at the top and for the other three ant species, at the bottom. The arrowhead indicates a transversion from C to G in the codon first position in *S. invicta* resulting in Ala (A), and not Pro (P) as in other ants, and the insertion of six additional nucleotides also extends the peptide length. There is a conservative replacement of R for H in *S. invicta* and F (TTT or TTC) is replaced with Y (TAC). The sequence for C-terminal α-amidation of Y or F is X-Gly-basic residue (R), and Gly provides the nitrogen for the same [70].

**Figure 3:**
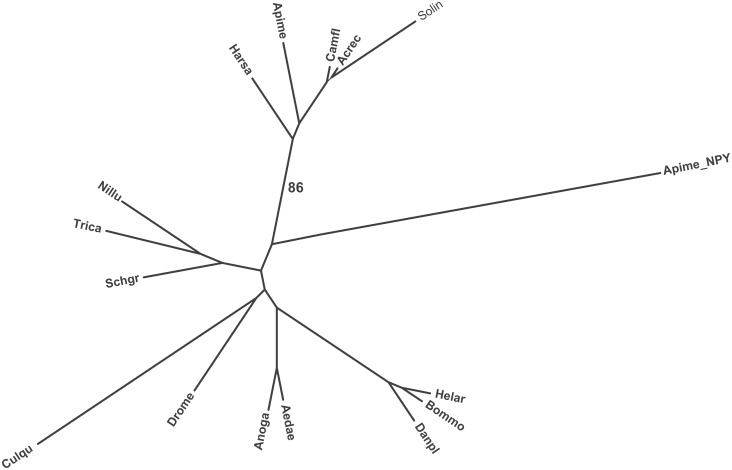
Phylogenic tree of insect short neuropeptide F prepropeptide sequences from different insect species. Amino acid sequences were analyzed by MEGA (version 5.05); the phylogenetic tree was constructed using the neighbor-joining method. The number 86 indicates the bootstrap confidence level. Abbreviations on branches correspond to the genus and species name, and these are identical to those described in the legend of Figure 2, with the addition of the Southern house mosquito, *Culqu* (*Culex quinquefasciatus* EDS32332.1) sNPF and the predicted sNPF from the ant species *Attce (Atta cephalotes), Linhu (Linepithema humile),* and *Pogba (Pogonomyrmex barbatus)*. The honey bee, *Apime* NPY (NP_001161192.1) was also included in the analysis.

Two cDNAs of *S. invicta* sNPF precursor (GenBank accession no# KJ812404 and KJ812405) were cloned from *S. invicta* mated queen brain, both encoding a protein of 112 amino acid residues (Figure 1). The longer cDNA is 3,732 bp and the shorter is 3,033 bp long. The 5′ untranslated region (UTR) (120 bp) and the ORF region are identical in both cDNA forms, differing only in the length of the 3′ UTR. The longer cDNA 3′ UTR is 3,273 bp and the shorter is 2,574 bp without poly (A) tail; the latter form arising from the usage of an earlier polyadenylation signal sequence (Figure 1, boxed). A Blast search with the deduced amino acid sequence predicted this protein as a sNPF from insects and, therefore, it was aligned to those. The alignment shows that arginine (R) at position 76, serine (S) at position 77, leucine (L) at position 84 and arginine (R) at position 85 are conserved in all aligned insect sequences (Figure 2A). The signal peptide prediction tool identified the prepropeptide signal peptide cleavage site between amino acid residues 24 (A) and 25 (T), yielding an 88-amino acid residue propeptide (Figure 1). The active peptides predicted were SLRSALAAGHLRYa (sNPF1) and SALAAGHLRYa (sNPF2) (Figure 1). These peptides end in an amidated tyrosine (Y) unlike phenylalanine (F) amine in other aligned insect species (Figure 2A). The fire ant sequence SLRSALAAGHLRYa is 30% identical to the genomic predictions of sNPF ligands from the other ant species SQRSPSLRLRFa (Figure S2). Closer analysis of the nucleotide sequences of sNPF ORFs from *S. invicta* and three ant species revealed that the sequences begin to differ just before those encompassing the active peptide. The amino acid sequences of the active peptide change by the additional downstream insertion of the nucleotides ‘TAGCTG’, which results in a sequence in frame. This results in the addition of two amino acid residues in the fire ant sNPF active peptide in comparison to the canonical sequence in other ants (Figure 2B). The prepropeptide sequences are somewhat similar in both percentage of sequence identity and structure within species of the same order (Figure 2A, Table S2), and this is especially true among the Hymenoptera (Figure S2, Table S2). Neighbor-joining phylogenetic analyses of the identified *S. invicta* sNPF prepropeptide with insects’ sNPFs and the honey bee long NPF (NPY) prepropeptides formed order-specific clusters (Figure 3), *S. invicta* sNPF grouping with the other hymenopterans sNPFs and honey bee long NPF (NPY). Similar to sNPF, the long NPF in most insect species ends in an amidated F, however, the honey bee NPF ends in an amidated Y (Table 1), and for this reason was included in the tree.

**Table 1:** Amino acid sequences of the ligands tested on the *Si*sNPFR-C6E8 cell line and *S. invicta* peptides EC_50_ values.

Peptide ligands	Amino acid sequences (a = amidated at C-terminus)	Activity (EC50)	% identity to *S*. *invicta*sNPF1
*S. invicta* sNPF1	SLRSALAAGHLRYa	3.2 nM	100
*S. invicta* sNPF2	SALAAGHLRYa	8.6 nM	100
Analogous sNP(F)	SLRSALAAGHLRFa	not active1 µM	92.3
*Drome* sNPF1	AQRSPSLRLRFa	not active1 µM	50
*Drome* sNPF2	WFGDVNQKPIRSPSLRLRFa	not active1 µM	40
*Drome* sNPF212–19	SPSLRLRFa*	not active1 µM	42.9
Mouse PYY	AKPEAPGEDASPEELSRYYASLRHYLNLVTRQRYa	not active1 µM	30.8
*Apime* NPY	EPEPMARPTRPEIFTSPEELRRYIDHVSDYYLLSGKARYa	not active1 µM	38.5

*S. invicta (Solenopsis invicta)*, Analogous sNP(F) (Analog of *S. invicta* sNPF1), *Drome (Drosophila melanogaster)*, *Apime (Apis mellifera)*.

*Sequence identical to *Apime* sNPF.

All other peptides tested were not active at 1 µM.

### The sNPF peptide signature was detected in eggs and brood

**Figure 4:**
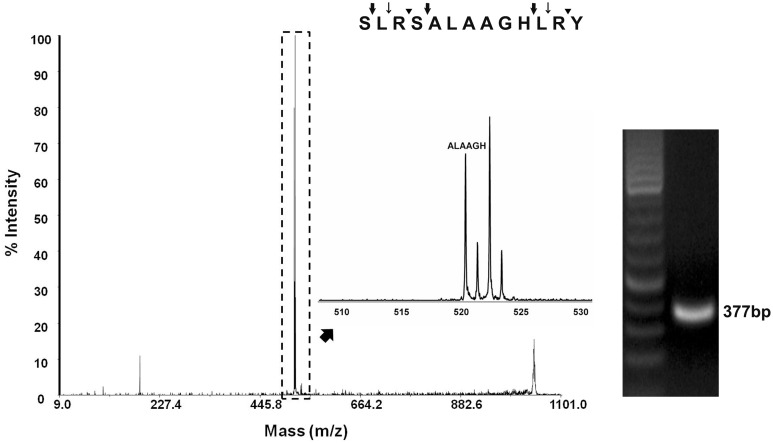
Detection of the fire ant sNPF (Partial sequence: ALAAGH) by MALDI-TOF MS. The Y-axis represents % intensity (as % of the highest charge detected) and the X-axis represents mass. The agarose electrophoresis analysis shows the amplified expected size band (377 bp) of the fire ant sNPF from the fire ant larvae cDNA. The peptide sequence analysis performed with the PeptideCutter tool at ExPASy Bioinformatics Resource Portal revealed several potential protease cleavage sites, such as those for proteinase K (thin arrows), thermolysin (thick arrows), and Arg-C proteinase, Clostripain, and trypsin (black arrow head). The detected fragment is predicted after thermolysis digestion.

MALDI-TOF MS detected a partial sequence of the sNPF peptide, ‘ALAAGH’, from the eggs and brood extract (Figure 4). To further support this finding, we also amplified a sNPF product from larvae cDNA. The expected sized band was obtained with a set of specific primers, SisNPF F2 and SisNPF R2 (Table S1) (Figure 4). The nucleotide sequence of the amplified PCR product was identical to that of the sNPF cDNA cloned from the fire ant queen brain. This verifies the expression of sNPF cDNA in brood, in agreement with the finding of the peptide signature. The analysis of the sNPF peptide sequence by PeptideCutter at ExPASy Bioinformatics Resource Portal predicts preferred cleavage sites for insect proteases such as trypsin and other endopeptidases of bacterial origin such as thermolysin (Figure 4). Two thermolysin sites flank the detected sequence ALAAGH, which is present in both sNPF1 and sNPF2.

### Development of a stable cell line expressing the HA-tagged *S. invicta* sNPF receptor

**Figure 5:**
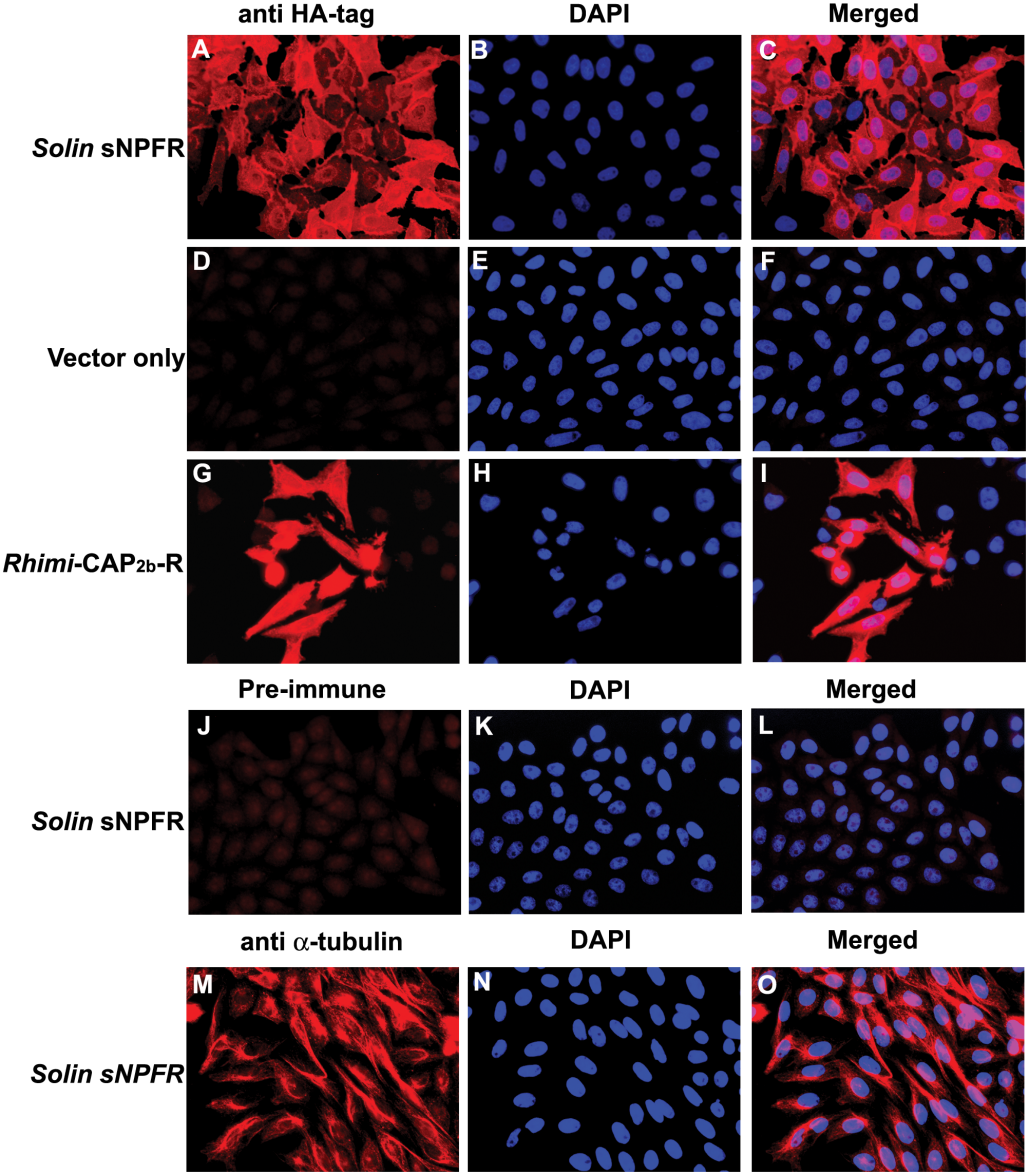
Detection of *Solenopsis invicta* short neuropeptide F receptor (*Solin* sNPFR) in stably transformed CHO-K1 cell line *Si*sNPFR-C6E8. The HA-tagged *Solin* sNPFR was detected by fluorescence immunocytochemistry using an anti-HA-tag antibody. The cell line names are shown on the left panel, and the antibody used labels the top of the first column. For each row, the images on the center show the nuclear staining with DAPI (blue) of the same cells on the left, and those on the right are merged images of the two previous. The HA-tag (red signal) is detected in *Solin* sNPFR cells (A–C) but not in the vector-only transformed cells (D–F). Rhimi-CAP_2b_-R cells (G–I) showing red signal were used as positive controls for the HA-tag labeling. No red signal was detected in *Solin* sNPFR cells incubated with pre-immune rabbit serum (J–L). An anti-α-tubulin antibody was used as positive control for the labeling of a cytoplasmic structural protein (M–O); the red pattern is different than for the HA-tag (contrast A and G to M). Scale bar, 50 µm.

**Figure 6:**
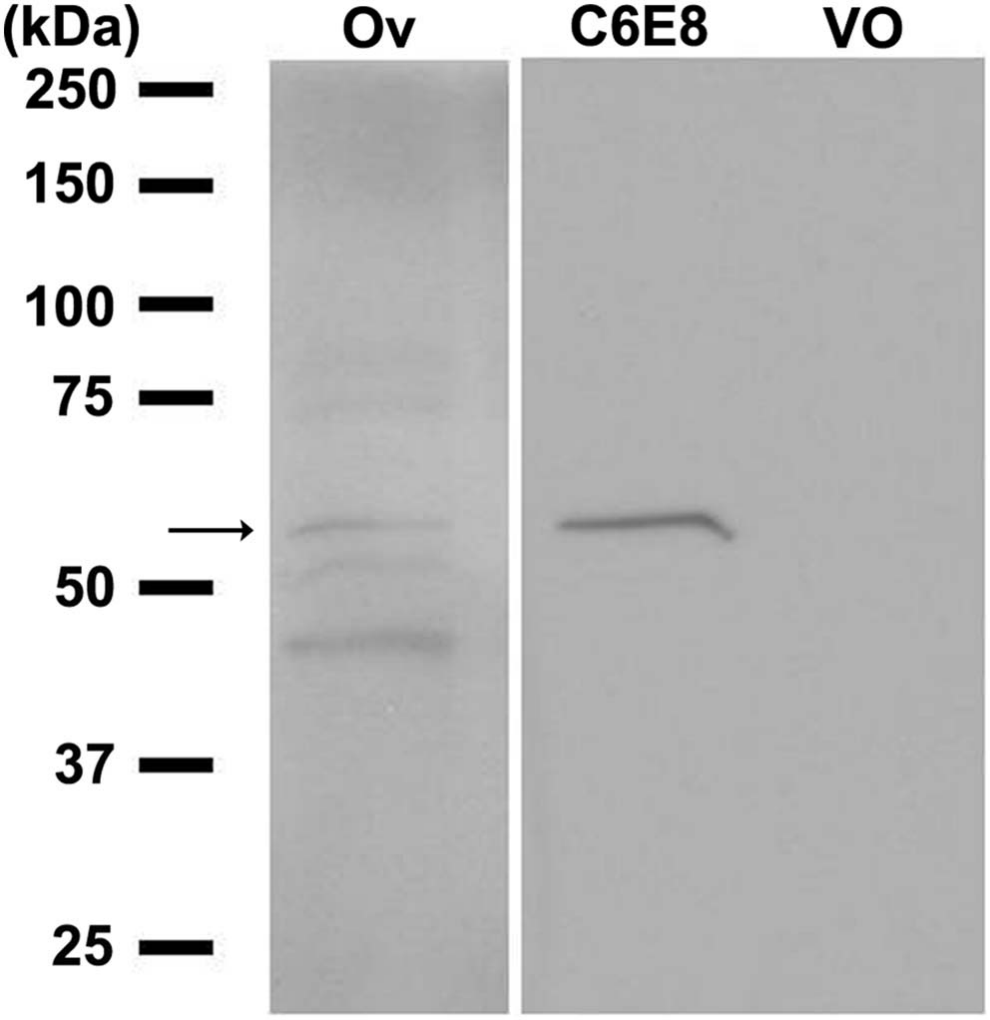
Detection of *S. invicta* sNPF receptor in cell membranes prepared from the *Si*sNPFR-C6E8 cell line by western blot analysis. Numbers in the left indicate the marker’s protein mass (kDa). Lane 1, the membrane fraction of the fire ant mated queen ovary (Ov) as a positive control; lane 2, the membrane fraction of *Si*sNPFR-C6E8 (C6E8) and lane 3, the membrane fraction of the vector-only (VO) (pcDNA3.1(-)) transfected CHO-K1 cells probed with the specific anti-sNPF receptor anti-peptide antibody [44]. Membrane protein (50 µg) was loaded in each lane. The antibody detects the 55 kDa receptor protein in lanes 1 and 2, as expected.

To deorphanize the *S. invicta* sNPFR, a CHO-K1 cell line stably expressing the receptor was selected. Expression of *S. invicta* sNPFR in the cell line *Si*sNPFR-C6E8 was verified by immunocytochemistry with an anti-HAtag antibody (Figure 5A–C). Negative controls, mock transfected (Figure 5D–F) and the stable cell line *Si*sNPFR-C6E8 incubated with pre-immune rabbit serum (Figure 5J–L) did not show any signal, as expected. The *Rhimi*-CAP2b-R #19 cell line which stably expresses a HA-tagged tick CAP_2b_ receptor [55], was used as positive control (Figure 5G–I). The *Si*sNPFR-C6E8 cell line was also stained with an anti-α tubulin antibody as an additional positive control; this showed the distinctive filamentous signal in the cell cytoplasm. Western blot analysis of the cell membrane preparation of *Si*sNPFR-C6E8 cells using a specific anti-peptide antibody against *S. invicta* sNPFR detected the specific band (55 kDa), verifying the expression of the full length receptor in the cells (Figure 6). A positive control with mated queen ovary tissue showed a band of the same size (Figure 6, lane 1, arrow).

### sNPY inhibited intracellular forskolin-stimulated cAMP in *Si*sNPFR-C6E8 cell line

**Figure 7:**
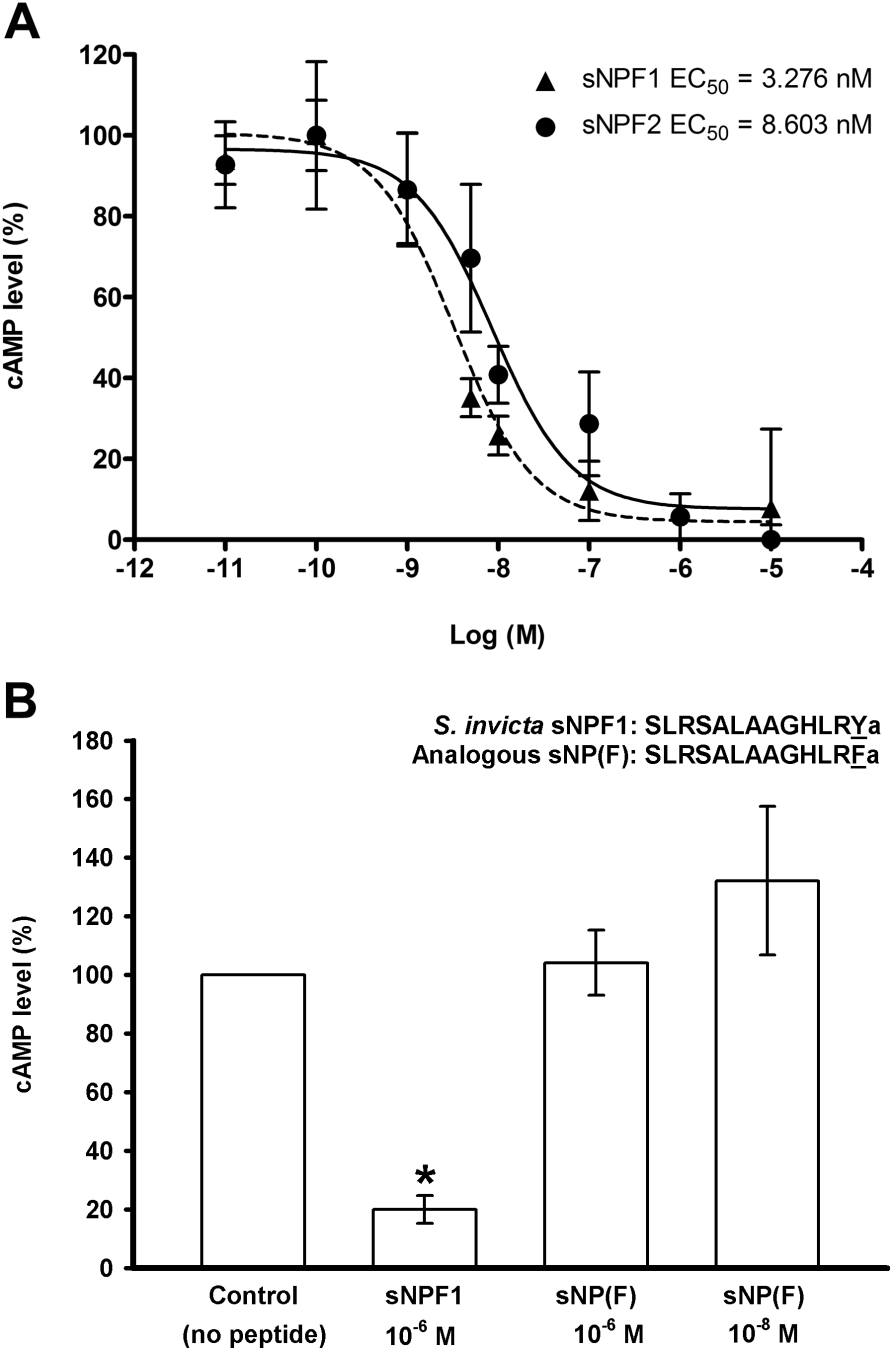
sNPF peptides inhibition of forskolin-stimulated cAMP accumulation in CHO-K1 cells stably transformed with the *S. invicta* sNPF receptor (*Si*sNPFR-C6E8). **A.** Independent dosage-response curves of *S. invicta* sNPF1 [SLRSALAAGHLRYa (13 a.a. residues); dashed line] and sNPF2 [SALAAGHLRYa (ten a.a. residues); solid line]. Cells were treated with a phosphodiesterase inhibitor previous to the simultaneous application of peptide and forskolin (10 µM), which was applied to elicit maximal cAMP production. The Y-axis represents the cAMP production as a percentage of the forskolin-stimulated maximum cAMP level, considered 100%. The X-axis shows the log molar (Log (M)) concentrations of peptides applied. Values indicate means ± S.E. of three independent biological replicates Half maximal effective concentration values (EC_50_ values) are given on the graph. **B.** In the fire ant sNPF1 sequence, the change of the C-terminal amidated residue ‘Y’ to ‘F’ [sNP(F)] eliminates the ligand activity on the receptor. *S. invicta* sNPF1 (1 µM) decreased cAMP as expected (*, significantly different at *p*≤0.05 level). Data were analyzed by ANOVA followed by Tukey’s multiple comparison test. Each value is the average of three biological replicates with the error bars showing standard deviation of the average for each treatment. The standard error of the analysis is 19.8.

A pilot study showed that *S. invicta* sNPF ligands, sNPF1 and sNPF2, did not stimulate production of intracellular cAMP in the *Si*sNPFR-C6E8 cell line. We then determined that both ligands inhibited forskolin-stimulated cAMP (Figure 7A). In addition, a concentration-dependent inhibition of intracellular cAMP was observed, with EC_50s_ values of 3.2 and 8.6 nM for sNPF1 and sNPF2, respectively (Table 1 and Figure 7A), although there is no significant difference between the two ligands curves (Figure 7A). To determine if the particular ending of the fire ant peptide (Ya) has functional significance, the analogous sNP(F) peptide was tested, in which the C-terminal amidated Y was replaced by amidated F, and showed no activity on the receptor (Figure 7B). Other peptides, *Drosophila* sNPFs, *Apime* NPY and mouse PYY also failed to change the cAMP production indicating they did not activate the sNPFR (Table 1).

## Discussion

We cloned two *S. invicta* sNPF cDNAs that differ only in the length of 3′ UTR but not in the 5′ UTR or coding region (Figure 1). The ORF of sNPF was flanked by a relatively long 3′ UTR (3.2 kb in the longer cDNA and 2.5 kb in shorter cDNA, which is identical to the longer cDNA). The length of the 3′ UTR is important in post-transcriptional regulation of gene expression such as mRNA degradation and stability, nucleo-cytoplasmic transport and mRNA localization, and regulation of translation initiation [58], [59]. Longer 3′ UTRs facilitate the formation of secondary structural intra-folding, which hinder miRNA-binding sites which might be accessible in shorter 3′UTRs, conferring greater stability to the mRNA [60]. The short cDNA arises from the utilization of an alternate poly (A) site (Figure 1). Multiple poly (A) signal sequences ‘AAUAAA’ were predicted on the 3′ UTR region of the sNPF longer cDNA. The differential expression of a number of genes that undergo alternative poly (A) site choice (polyadenylation) or splicing competition could be regulated at the level of tissue-specific polyadenylation factors [61], [62]. The use of alternative poly (A) sites can impact the final amount of protein product per unit precursor of RNA transcribed [61]. It would be interesting to know if the length of the sNPF 3′ UTR varies also according to fire ant caste, developmental stage and/or neuron-specific manner. Since we cloned it from queens, it is possible that 3′ UTR length could be neuron-specific.

In fruit fly, the African malaria mosquito, the yellow fever mosquito, and the silkworm *Bombyx mori*, the sNPF gene encodes a single prepropeptide which is processed to yield four to five individual sNPFs [21], [24]–[26], [30] (Table S2). The *S. invicta* sNPF gene encodes a prepropeptide which may yield, only alternatively, two possible active sNPF peptides that may differ only in length by three amino acid residues at the N-terminal region (Figure 2A). Alignment of sNPF prepropeptide of different insect species revealed that fire ant prepropeptide is more similar to those of other social insects such as other ant species (from which genomic information allowed sNPF peptide prediction [63]), and the honey bee than to solitary insects in the Diptera, Coleoptera and Lepidoptera (Table S2). In most arthropods studied so far, sNPFs are 6–11 amino acid residues in length with the C-terminal consensus sequence xPxLRLRFamide [21]. Interestingly, *S. invicta* sNPF has an amidated tyrosine at the C-terminus, which is more similar to the honey bee “long NPF” (which ends in Y) (Table 1) and the vertebrate NPY. Phylogenetic analysis of the prepropeptide amino acid sequence showed that sNPF prepropeptides are different among different insect orders; it is known that regions with high variability indicate fast peptide sequence evolution [30], so it may be that sNPFs are evolving rapidly in insects.

Detection of the signature sequence of *S. invicta* sNPF from fire ant eggs and brood (Figure 4) supports the uniqueness of the amino acid sequence of the *S. invicta* sNPF compared to the other insect species (Figure 2; Figure S2). sNPFs are also present in the central nervous system of *Drosophila* larvae [35]. In fire ant colonies, fourth instar larvae have a critical role in dietary protein digestion, and peptides so processed are shared by the colony members, mainly given to other larvae and queen, through trophalaxis. These larvae perform extraoral digestion in addition to digestion that occurs in their gut. Therefore, is very likely that these abundant proteases may have digested the propeptide and the active ligand during their homogenization, because we did not add protease inhibitors to the homogenate. This explains the detection of only a partial sNPF sequence. A search of the fire ant genome for other potential proteins exhibiting the detected sequence ALAAGH did not yield any protein with that motif, although proteins with the motifs LAAGH or ALAA were found, further supporting the detected fragment corresponds to the sNPF peptide and that the fire ants translate the sNPF cDNA. We attempted to detect sNPF from intact brain of the newly mated queen. Availability of the samples was the main limiting factor for this particular experiment. The problem associated with detection of low abundant peptides because of limited body and organ size, physiological stage of the insect species using mass spectrometry technologies has been described [64].

In western blots of the *Si*sNPFR-C6E8 cell line, the molecular weight of the receptor band is 55 kDa (higher than expected mass of ∼46 kDa) when probed with the specific anti-sNPF receptor antibody (Figure 6). A similar analysis using the anti-HA tag antibody also showed a single band of the same size (not shown). In the mated queen ovary the receptor also runs at ∼55 kDa [44] (Figure 6). Immunocytochemistry showed that the majority of the cells expressed the recombinant receptor. In the membrane-proximal C-termini of many G protein-coupled receptors the sequence F(X_6_)LL (where X can be any residue, and L is leucine or isoleucine) is highly conserved and functions as a common motif mediating receptor transport from the endoplasmic reticulum to the cell surface [65]. In the *S. invicta* sNPFR this motif is represented by the sequence ‘FRKEFQQIL’ which is likely involved in the expression of *S. invicta* sNPFR on the cell surface (Figure S1). Both ligands (sNPF1 and sNPF2) activate the *S. invicta* sNPFR and reduce intracellular cAMP; moreover, a concentration-dependent inhibition of forskolin-stimulated cAMP was also observed with both. This indicates that the *S. invicta* sNPFR is a Gαi protein binding GPCR in CHO-K1 cells. The only other two similar studies in deorphanization of sNPF receptor in insects are for solitary insects, mosquito and locust, and these also showed a concentration-dependent inhibition of forskolin stimulated cAMP in CHO-K1 cells [27], [66]. The EC_50_ value for the mosquito sNPF in this recombinant system was reported as 1.5 nM [66], which is comparable with the EC_50_ values of 3.276 nM and 8.603 nM for *S. invicta* sNPF1 and sNPF2, respectively. Whereas the EC_50_ values were 76.3–95.5 pM for the inhibition of intracellular cAMP by locust sNPF on the cognate receptor expressed in HEK 293T cells [27]. In contrast with the above mentioned results, in *Drosophila* BG2-C6 cells which are of neuronal origin and endogenously express the sNPF receptor, the application of sNPF increases cAMP in a dosage-dependent manner [36], [67]. However, a genetically encoded FRET-based sensor for cAMP showed that sNPF causes a decrease in cAMP in *Drosophila* 3^rd^ instar larval motor neuron [68], suggesting the presence of tissue (or neuron)-specific sNPF downstream pathways.

Two NPY-family receptors, the NPF- and sNPF-receptor, have been identified in insects, with respective ligands being NPF and sNPF, respectively. Extensive searches in the honey bee and wasps genomes failed to identify the NPF receptor gene, indicating the loss of the gene in early hymenopteran evolution [41]. However, in the honey bee genome two NPY-like peptides, NPF (which ends in an amidated Y) and sNPF (ends in an amidated F) are present [32]. This finding indicates that the NPY system in the honey bee is functionally represented by the sNPF signaling system. It is unknown if the NPY peptide activates the sNPF receptor in honey bee and this would be interesting to know to determine if two peptides converged on the same signaling receptor, perhaps signaling through different G-proteins. We thus speculated that if the honey bee has one sNPF receptor but two related peptides (sNPF and NPY), perhaps the fire ant sNPF receptor could be a “permissive receptor” being activated by other NPY-like ligands. However, neither the honey bee NPY (long NPF; 39 amino acid residues) nor vertebrate PYY (34 amino acid residues) activated the fire ant receptor despite the similarity of these ligands to *S. invicta* sNPF in having an amidated tyrosine at the C-terminus and other sequence similarities (Table 1, underlined residues). Based on these findings it appears that social insects have significant differences with respect to solitary insects in neuropeptide networks controlling the propensity to food searching and acquisition [69] and of sensing their nutritional status [45]. In summary, the *S. invicta* sNPFR has been deorphanized by identifying a non-canonical sNPF peptide sequence ending at amidated ‘Y’ from *S. invicta*, and this residue is critical for receptor activation and decrease in intracellular cAMP production. These conclusions are supported by genomic, RNAseq and cloning data as well as detection of a signature peptide fragment by MALDI-TOF MS and by functional cAMP assays. The receptor exhibits high selectivity for *S. invicta* sNPF1 and sNPF2 which have equal potency, in the nanomolar range.

## Supporting Information

Figure S1 Corrected open reading frame sequence of *S. invicta* sNPF receptor.
(TIFF)

Figure S2 Alignment of sNPF pre-propeptide sequences from the seven ant species for which genome sequences are available. Identical residues to the fire ant sequence are on black background. The active sNPF peptide for all species is boxed and the sequence SLRSALAAGHLRYa is 30% identical to the genomic predictions of sNPF ligands (SQRSPSLRLRFa) from the other ant species. *Solin, Solenopsis invicta; Attce, Atta cephalotes; Acrec, Acromyrmex echinatior; Camfl, Camponotus floridanus; Harsa, Harpegnathos saltator, Linhu, Linepithema humile, Pogba, Pogonomyrmex barbatus*.
(TIFF)

Table S1 Primer information.
(DOCX)

Table S2 Percentage of amino acid sequence identity between the fire ant sNPF pre-propeptide and those from the respective insect species listed. Insect orders of species listed in descending order are: Hymenoptera (first 7 species including 6 ants), Orthoptera, Coleoptera, Diptera (3 species), Hemiptera and Lepidoptera (3 species).
(DOCX)
