# Supplementary figures and images for: The Red Imported Fire Ant (Solenopsis invicta Buren) Kept Y not F: Predicted sNPY Endogenous Ligands Deorphanize the Short NPF (sNPF) Receptor
Source: PLoS One. 2014 Oct 13;9(10):e109590. doi: 10.1371/journal.pone.0109590 (PMC4195672; doi:10.1371/journal.pone.0109590)

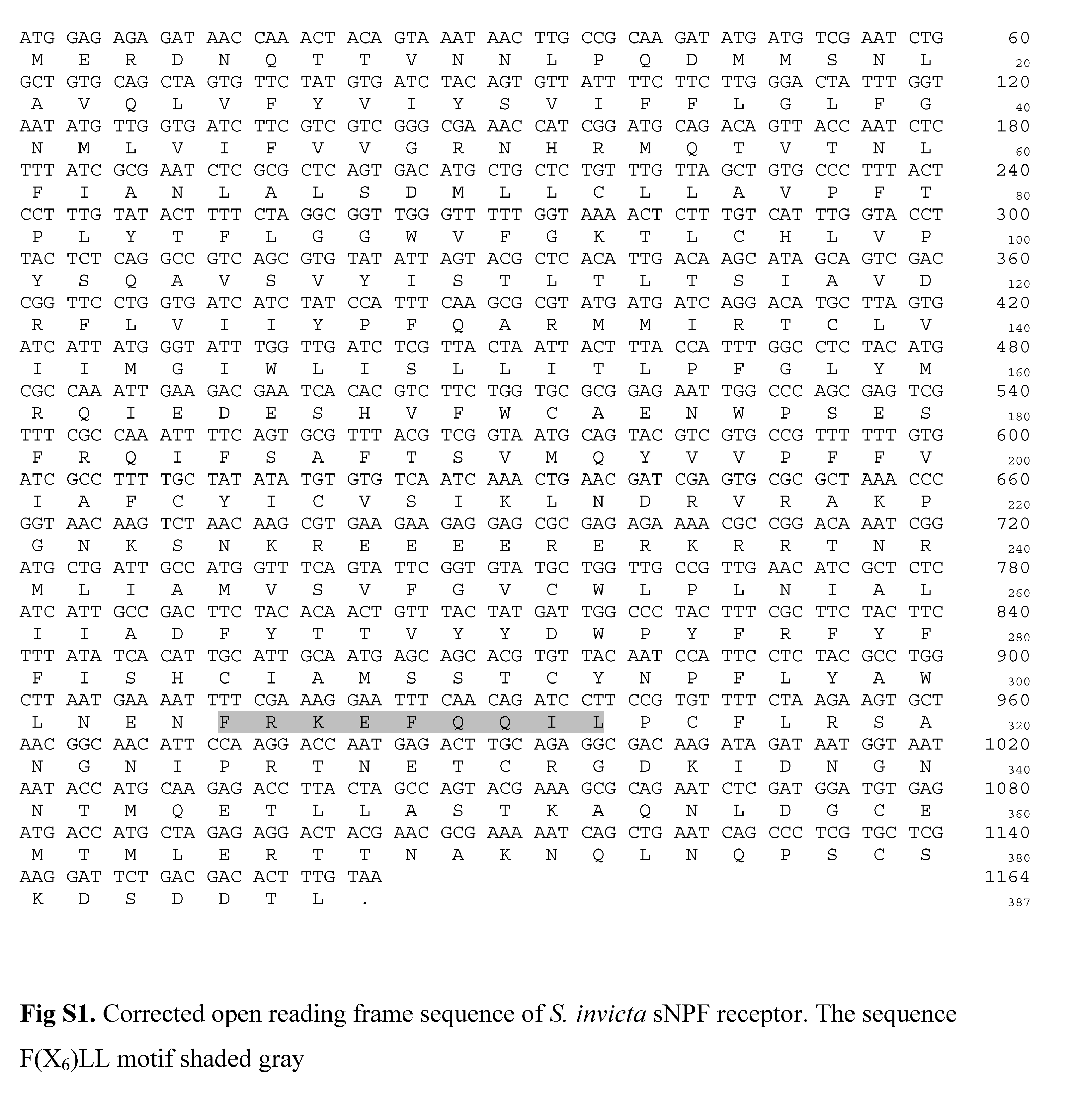

Supplement: Figure S1 — Corrected open reading frame sequence of S. invicta sNPF receptor. (TIFF) [file pone.0109590.s001.tiff]
