# Supplementary material for: The Red Imported Fire Ant (Solenopsis invicta Buren) Kept Y not F: Predicted sNPY Endogenous Ligands Deorphanize the Short NPF (sNPF) Receptor
Source: PLoS One. 2014 Oct 13;9(10):e109590. doi: 10.1371/journal.pone.0109590 (PMC4195672; doi:10.1371/journal.pone.0109590)
Supplement: Table S1 — Primer information. (DOCX) [file pone.0109590.s003.docx]

| Primer name | Primer sequence |
| --- | --- |
| **Primers for sNPF cloning** | |
| Si sNPF-F1 | 5’ CTGAGACTAACGCTCAAGAATTCGAACCCG 3’ |
| Si sNPF-R1 | 5’ TCAGATGATAACACCGCCATGGTCGTACAG 3’ |
| 5' SI sNPF-R1 | 5' CGGAAAGGTGTCCCTGGAACAGCCTCACCGA 3' |
| 3' Si sNPF-F1 | 5' CTTGACCAGTTTGTCGCCTCGTAATTAGCC 3' |
| 3' Si sNPF-NF1 | 5' CTGTACGACCATGGCGGTGTTATCATCTGA 3' |
| **Primers for expression in mammalian cell** | |
| Primer#129 | 5’-AGACTCGAGGCCACC**ATG**GCC***TACCCCTACGACGTG***-3’ |
| sNPFR-f1 | 5’-GCC***TACCCCTACGACGTGCCCGACTACGCC***GAG**-**3’ |
| sNPFR-f2 | 5’ ***GTGCCCGACTACGCC***GAGAGAGATAACCAAACT-3’ |
| sNPFR-r1 | 5’- AATCGTGAATTCCAATGACAACAGGTCGCT 3’ |
|  | **Primers for the amplification of sNPF from larvae** |
| Si sNPF-F2 | 5’ CCGAGGACAGAGGAGGCCATGTATGCTAAA 3’ |
| Si sNPF-R2 | 5’ CCCTGGAACAGCCTCACCGATTAATTGTTG 3’ |

**Table S1**

Foot Note:

*Xho*I restriction site underlined, the Kozak sequence shaded, a start codon bold and underlined, and a sequence of HA-tag bold and italics, and the *Eco*R1 restriction site double underlined
