# Supplementary material for: The Red Imported Fire Ant (Solenopsis invicta Buren) Kept Y not F: Predicted sNPY Endogenous Ligands Deorphanize the Short NPF (sNPF) Receptor
Source: PLoS One. 2014 Oct 13;9(10):e109590. doi: 10.1371/journal.pone.0109590 (PMC4195672; doi:10.1371/journal.pone.0109590)
Supplement: Table S2 — Percentage of amino acid sequence identity between the fire ant sNPF pre-propeptide and those from the respective insect species listed. Insect orders of species listed in descending order are: Hymenoptera (first 7 species including 6 ants), Orthoptera, Coleoptera, Diptera (3 species), Hemiptera and Lepidoptera (3 species). (DOCX) [file pone.0109590.s004.docx]

**Table S2:** Percentage of amino acid sequence identity between the fire ant sNPF pre-propeptide and those from the respective insect species listed. Insect orders listed are: Hymenoptera (first 7 species including 6 ants), Orthoptera, Coleoptera, Diptera (3 species), Hemiptera and Lepidoptera (3 species).

| Insect species | % identity | Insect species | % identity |
| --- | --- | --- | --- |
| *Acromyrmex echinatior,* leaf-cutter ant | 73.8 | *Tribolium castaneum,* flour beetle | 21.8 |
| *Camponotus floridanus,* carpenter ant | 73.8 | *Anopheles gambiae,* malaria mosquito | 23.9 |
| *Pogonomyrmex barbatus,* red harvester ant | 59.7 | *Aedes aegypti,* yellow fever mosquito | 22.8 |
| *Linepithema humile,* Argentine ant | 58.9 | *Drosophila melanogaster,* fruit fly | 24.7 |
| *Atta cephalotes,* leaf-cutter ant | 49.5 | *Nilaparvata lugens,* planthopper | 21.2 |
| *Apis mellifera,* honey bee | 48.1 | *Helicoverpa armigera,* cotton bollworm | 24.5 |
| *Harpegnathos saltator*, basal ant | 43.3 | *Danaus plexippus,* monarch butterfly | 25.5 |
| *Schistocerca gregaria,* locust | 26.3 | *Bombyx mori,* silkworm | 24.5 |
